# Alumni Meeting

**Published:** 1871-06

**Authors:** 


*Alumni Meeting.*

AMERICUS, April 13, 1871.

We, a portion of the Alumni of Atlanta Medical College, desirous of having an annual reunion in the halls of our Alma Mater, take this method of calling the attention of our classmates to this subject.

We propose that a meeting be had on the first day of September next, at the Annual Commencement of the Institution.

All Alumni of the College, wherever they may reside, are cordially invited to attend, and will have tendered them the hospitalities of the Faculty of the College on the occasion.

| | | |
|---|---|---|
| W. H. PHILPOT, | E. L. CONNALLY, | A. H. SHI, |
| J. C. JOHNSON, | W. O'DANIEL, | T. J. MITCELL, |
| T. R. COOK, | F. M. HUNT, | W. N. JUDSON, |
| H. PERDUE, | S. A. WILSON, | J. M. DANIEL. |

